# Microstructure and Properties of Electrodeposited Nanocrystalline Ni-Co-Fe Coatings

**DOI:** 10.3390/ma14143886

**Published:** 2021-07-12

**Authors:** Piotr Ledwig, Malgorzata Kac, Agnieszka Kopia, Jan Falkus, Beata Dubiel

**Affiliations:** 1 Faculty of Metals Engineering and Industrial Computer Science, AGH University of Science and Technology, al. Adama Mickiewicza 30, 30-059 Kraków, Poland; kopia@agh.edu.pl (A.K.); jfalkus@agh.edu.pl (J.F.); 2 Institute of Nuclear Physics, Polish Academy of Sciences, ul. Radzikowskiego 152, 31-342 Kraków, Poland; malgorzata.kac@ifj.edu.pl

**Keywords:** electrodeposition, Ni-Co-Fe coatings, nanocrystalline alloys, magnetic properties, corrosion properties, microstructure characterization

## Abstract

Materials based on Ni-Co-Fe alloys, due to their excellent magnetic properties, attract great attention in nanotechnology, especially as candidates for high-density magnetic recording media and other applications from spintronic to consumer electronics. In this study, Ni-Co-Fe nanocrystalline coatings were electrodeposited from citrate-sulfate baths with the Ni^2+^:Co^2+^:Fe^2+^ ion concentration ratios equal to 15:1:1, 15:2:1, and 15:4:1. The effect of the composition of the bath on the morphology, microstructure, chemical composition, microhardness, and magnetic properties of the coatings was examined. Scanning (SEM) and transmission (TEM) electron microscopy, X-ray diffractometry (XRD), and energy dispersive X-ray spectroscopy (EDS) were used to study surface morphology, microstructure, chemical, and phase composition. Isothermal cross-sections of the Ni-Co-Fe ternary equilibrium system for the temperature of 50 °C and 600 °C were generated using the FactSage package. Magnetic properties were analyzed by a superconducting quantum interference device magnetometer (SQUID). All the coatings were composed of a single phase being face-centered cubic (fcc) solid solution. They were characterized by a smooth surface with globular morphology and a nanocrystalline structure of grain diameter below 30 nm. It was determined that Ni-Co-Fe coatings exhibit high hardness above 4.2 GPa. The measurements of hysteresis loops showed a significant value of magnetization saturation and small coercivity. The microstructure and properties of the obtained nanocrystalline coatings are interesting in terms of their future use in micromechanical devices (MEMS).

## 1. Introduction

Electrodeposition of nanostructured materials plays an essential role in nanotechnology. One application of this technique is the fabrication of magnetic parts for MEMS. The selection of appropriate deposition conditions to obtain nanocrystalline coatings with smooth surface morphology, high microhardness, low magnetic coercivity, high magnetic saturation, good corrosion, and wear resistance is crucial for good quality magnetic MEMS components.

Ferromagnetic materials used in nanotechnology contain Ni, Fe, and Co 3*d* transition metals. Among the Ni-Fe alloys, the most widely used is permalloy, Ni_81_Fe_19_, characterized by low coercivity and saturation flux density of about 1 T. Increasing Fe content allows an increase in the magnetic saturation value. In turn, binary Co-Fe alloys show the highest magnetic flux density among the alloys of the iron group, reaching over 2.4 T, but also exhibit high magnetic coercivity, high internal stresses, brittleness, and poor corrosion resistance [1]. The improvement of their properties might be carried out by the addition of alloying elements such as Ni or Cu [1,2]. Accordingly, ternary Ni-Co-Fe alloys are of great interest in recent years due to their superior soft magnetic properties [3,4,5,6,7,8,9]. Moreover, Ni-Co-Fe alloys are applicable as anticorrosive or antireflective coatings, catalysts, and very low thermal coefficient materials [5,10,11]. Many methods such as metal casting, chemical synthesis [12] are used for the production of bulk ternary Ni-Co-Fe alloys, in turn for thin coatings sputtering [13], molecular beam epitaxy [14], or electrodeposition are used [2,15,16,17]. Electrodeposition is often preferred over vacuum processes due to its low cost and high effectiveness. Thin coatings obtained by electrodeposition are characterized by low surface roughness, nanocrystalline structure, and often better corrosion and mechanical properties than bulk materials.

A good example of electrodeposited Ni-Co-Fe alloys is Co_65_Ni_12_Fe_25_, achieved by Osaka et al. [16], which is characterized by a very low coercivity value of 1.2 Oe and high magnetic flux density in the range 2.0–2.1 T. Additionally, Liu et al. electrodeposited Ni-Co-Fe alloy with coercivity about 1 Oe [18].

The control of the chemical composition of electrodeposited Ni-Co-Fe alloys is important because even a small change in concentration of alloying elements can lead to significant changes in the microstructure and properties. However, it is not easy because the electrodeposition of chemical elements from the iron group is anomalous [8,19]. The mechanism of anomalous deposition is widely known and relies on the increase in hydroxyl ions concentration and formation of the metal hydroxides surface layer on the cathode, which suppresses the reduction of less electronegative ions [19].

Electrodeposition of ternary alloys is usually carried out from baths based on sulfates or chlorides with sulfur-containing organic additives, such as saccharin [11,20], thiourea [20], or sodium laurylosulfate (SLS) [21], which have the role of refining the nanostructure of electrodeposited coatings. Electrodeposition conditions and bath composition must be appropriately selected to obtain nanocrystalline coatings with low internal stresses and low content of impurities [1,22]. Moreover, to obtain superior soft magnetic properties, the grain size should be as small as possible [23].

Although electrodeposited Ni-Co-Fe coatings are good candidates for applications in magnetic MEMS parts, systematic studies of deposition, microstructure, and properties are lacking. The few reports describe the influence of the chemical composition on the surface morphology [6,11,24]. It is demonstrated that the higher Co content promotes the formation of the needle-like morphology, while the higher nickel favors globular or polyhedral morphology. However, the correlation between the surface morphology, phase composition, and roughness parameters is missing. There is also a lack of information on TEM investigations of the microstructure; hence, the relationship between the microstructure, mechanical and magnetic properties, and corrosion resistance of Ni-Co-Fe coatings has not been established.

An optimal combination of good mechanical properties, corrosion resistance, adhesion, and magnetic properties is required for applications in magnetic MEMS devices [25]. Therefore, the goal of this study is to obtain electrodeposited nanocrystalline Ni-Co-Fe coatings with the properties required for good-quality magnetic MEMS components. To achieve this, the deposition conditions were selected, and in-depth studies of the surface morphology and roughness, microstructure, microhardness, magnetic properties, and electrochemical corrosion resistance of the coatings were performed.

## 2. Materials and Methods

In this work, the ternary Ni-Co-Fe coatings were electrodeposited from sulfate-citrate baths. The process was carried out in a three-electrode system, where working, counter, and reference electrodes were, respectively, copper plate, platinum plate, and saturated Ag/AgCl electrode. Electrodeposition was performed in citrate-sulfate baths using potentiostat/galvanostat PGSTAT 302N (Metrohm, Herisau, Switzerland). The chemical composition and parameters of the process are collected in Table 1.

The baths characterized by Ni^2+^:Co^2+^:Fe^2+^ ratios equal to 15:1:1, 15:2:1, and 15:4:1 were marked as NCF1, NCF2, and NCF3, then the same names were used for the coatings deposited from them.

Before the electrodeposition, the copper substrates were ground on water papers with gradation up to 2000. To remove pollutions and degrease substrate surface two-step cleaning process was used. Firstly, copper plates were cleaned using a mixture of distilled water and ethanol in ultrasound cleaner and before electrodeposition in acetone. No chemical surface treatment was applied to the substrate. Rectangular copper plates with dimensions equal to 25 mm × 15 mm × 1 mm were used, while the area of the coatings was reduced to a square with a side of 15 mm. The deposition was carried out for 20 min to obtain a thickness of about 10 µm.

The surface morphology and the microstructure of the coatings were investigated using an Inspect S500 SEM of FEI (Hillsboro, OR, USA) with secondary electrons (SE) and backscattered electrons (BSE) contrast. Microanalysis of chemical composition was performed using EDS with an Octane Elect detector (EDAX Ametek, Berwyn, IL, USA). For each coating, the composition was determined based on the quantitative analysis of a sum spectrum collected for a total area of over 5000 µm^2^ of the plan-view specimen at an accelerating voltage equal to 15 kV.

Roughness parameters were measured by the Wyko NT930 optical profilometer (Veeco, Plainview, NY, USA) using vertical scanning interferometry (VSI) mode. The roughness parameters of each sample were measured on a total area equal to 1.17 mm^2^. Roughness parameters R_a_, R_q_ and R_t_ are defined as the arithmetical mean deviation of the assessed profile, root mean squared roughness, and the maximum height of the profile, respectively.

The microstructure of coatings was investigated by TEM JEM-2010 ARP (Jeol, Tokyo, Japan). Image analysis and stereological measurements of equivalent circle diameter (ECD) of grains were performed using ImageJ 1.50i software (ImageJ, Bethesda, MD, USA).

The phase composition of coatings was investigated using selected area electron diffraction (SAED) patterns and XRD. The JEMS program by P. Stadelmann (JEMS-SWISS, Jongny, Switzerland) was used to solve the SAED patterns. Phase crystallographic data was taken from the Inorganic Material Database [26].

XRD patterns were acquired using a Panalytical Empyrean DY 1061 (Malvern Panalytical, Almelo, The Netherlands) diffractometer in Bragg–Brentano geometry with Cu Kα radiation (λ = 0.154 nm). A PDF-4+ database (ICDD, USA) was used for phase identification. The crystallite size was estimated based on the Scherrer equation using full-width half maxima (FWHM) for (111) peaks.

Thermodynamical calculations for equilibrium conditions of the Ni-Co-Fe system were performed using the FactSage package (GTT-Technologies, Herzogenrath, Germany) and SGTE database 2017 (Scientific Group Thermodata Europe, St Maintint d’Heres, France). Isothermal cross-sections for the temperature of 50 °C and 600 °C were generated. The temperature of 50 °C is the temperature of the electrolyte used for deposition, while 600 °C corresponds to a homologous temperature value 0.49 ± 0.01 for ternary Ni-Co-Fe alloys.

The microhardness measurements were carried out using a Tukon 2500 microhardness tester (Wolpert Wilson, Nirwood, OH, USA) with the Knoop intender under a load equal to 0.1 N. At least eight measurements were completed for each coating. The achieved hardness values were converted from HK to GPa.

Magnetic properties were measured using a SQUID magnetometer (MPMS, Quantum Design North America, San Diego, CA, USA) by applying an external field of up to 4 T in-plane and out-of-plane of the sample. The measurements were carried out at room temperature. For SQUID investigations, samples with a coating thickness of about 1 μm were prepared.

To determine the corrosion resistance of Ni-Co-Fe coatings, polarization test and impedance spectroscopy (EIS) measurements were performed with the use of potentiostat/galvanostat Autolab PGSTAT 302N (Metrohm, Switzerland) working in a three-electrode system. The corrosion potential (E_corr_) and corrosion current (i_corr_) were calculated based on the Tafel extrapolation method. To determine the equivalent circuits, Nova 2.0 software was used. The average corrosion speed (V_corr_) was calculated using Equation (1):

(1) $$V_{\mathrm{corr}}=\frac{h}{t}=\frac{K_{\mathrm{NCF}}j_{\mathrm{corr}}}{\mathrm{zF}\rho },$$

where: K_NCF_—the electrochemical equivalent of Ni-Co-Fe alloy, j_corr_—corrosion current density, ρ—the density of Ni-Co-Fe alloy, z—valence, and F—Faraday constant.

## 3. Results and Discussion

### 3.1. Chemical Composition

The use of electrolytic baths with Ni^2+^: Co^2+^:Fe^2+^ ratios equal to 15:1:1, 15:2:1, and 15:4:1 led to electrodeposition of coatings with a Ni_52_Co_23_Fe_25_ (NCF1), Ni_46_Co_37_Fe_17_ (NCF2), and Ni_30_Co_56_Fe_14_ (NCF3) composition. In electrodeposited coatings, the Co:Fe ratio was similar to the Co^2+^:Fe^2+^ ions concentration ratio in the bath. However, Co^2+^ and Fe^2+^ ions were preferentially reduced before Ni^2+^; thus, the anomalous electrodeposition took place. The anomalous character of electrodeposition of Fe and Co ions before Ni is a well-known phenomenon [15] and was also described in our previous works on electrodeposition of binary Ni-Co [27] and Ni-Fe alloys [28] from citrate-sulfate baths. Differences between Fe and Co concentrations in coatings were small, but a slightly higher concentration of Fe was achieved. Kinetics of Fe and Co ions deposition depended on the chemical composition of the bath, especially pH of the bath, anions addition, or electrodeposition parameters. The chemical composition of the coatings obtained in our study was in good agreement with the results of Zhang et al. [29], who observed that in most examined conditions, Fe ions reduced at a higher rate than Co ions.

Although electrolytic baths components were sulfur-containing organic compounds, such as saccharin and SLS, the EDS microanalysis did not show any contamination in the electrodeposited coatings.

### 3.2. Surface Morphology and Roughness

**Figure 1:**
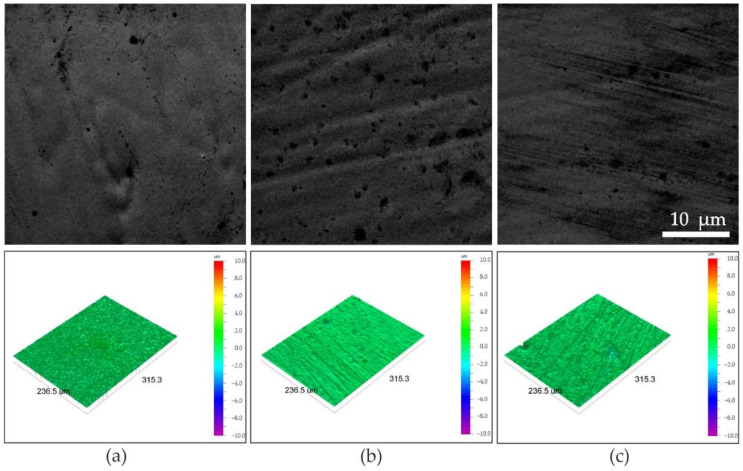
SEM SE images of surface morphology and roughness maps of (**a**) NCF1, (**b**) NCF2, (**c**) NCF3 coatings.

Figure 1 shows SEM images of surface morphology. All coatings were characterized by smooth surface morphology with small nodules. Except for single micrometric size nodules and characteristic patterns formed by gas bubbles, surface morphology was homogeneous. Characterization of the coatings’ surface also included the roughness measurements. The values of R_a_, R_q_, and R_t_ parameters are collected in Table 2. All the coatings were characterized by a smooth surface with R_a_ parameters values below 0.21 μm. The lowest surface roughness was achieved for NCF2 coating. The values of the R_t_ parameter were similar for all coatings. Low values of roughness parameters are in good agreement with surface morphology observations in SEM and correspond to a low number of irregularly shaped surface islands, discontinuities, or pores. It was found that the roughness parameters of Ni-Co-Fe coatings were in a similar range as for binary Ni-Fe [28] and Ni-Co coatings [27].

It is well known that chemical and phase composition has a significant influence on surface morphology. Kocknar et al. for ternary Ni-Co-Fe alloys with a high concentration of Co observed needle-like surface morphology, while the globular morphology appeared for coatings with higher Ni content [6]. In turn, Kim et al. observed the polyhedral surface morphology of Ni-Co-Fe alloys [11]. The small diameter of morphological structures might be caused by organic additives, such as saccharin, which have leveling and grain refinement effects in electrodeposited Ni and Ni-based alloys. Saccharin is a very common additive in electrolytic baths used for deposition of Ni coatings, but there are only a few reports of the use of saccharin in Ni-Co-Fe electrodeposition [11,24].

In previous work, we observed the globular surface morphology of Ni-Fe coatings and globular or globular-needle morphology of Ni-Co coatings [27]. The presence of needle-like morphology often indicates a mixed fcc and hexagonal close-packed (hcp) microstructure [11,30,31]. We did not observe any needle morphology in this experiment, indicating that hcp Co-based phases were not formed in ternary Ni-Co-Fe coatings.

### 3.3. Phase Composition

**Figure 2:**
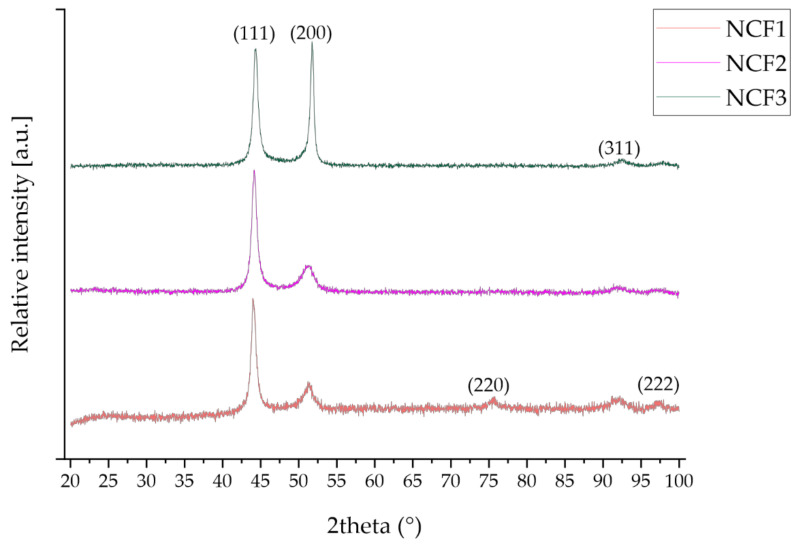
XRD spectra of ternary Ni-Co-Fe coatings.

**Figure 3:**
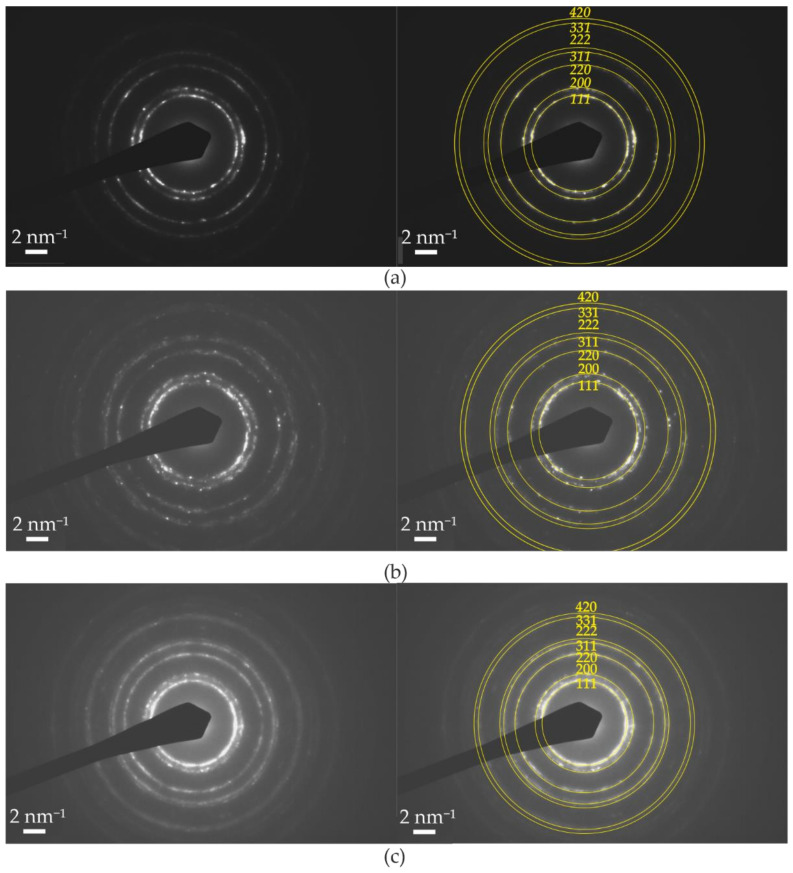
SAED patterns of (**a**) NCF1, (**b**) NCF2, and (**c**) NCF3 ternary Ni-Co-Fe coatings with solutions for γ Ni standard.

The phase composition of the coatings was investigated using XRD and SAED patterns given in Figure 2 and Figure 3. The XRD results are collected in Table 3.

From XRD patterns, only one phase was identified using a standard of Co_0.2_Fe_0.4_Ni_0.4_ solid solution with fcc structure [12]. The strong peaks from (111) and (200) crystal planes were noticed. SAED patterns of all coatings consisted of continuous rings assigned using the crystallographic data of γ-Ni. On this basis, it was stated that the analyzed phase is the γ Ni-based solid solution, which is in line with the XRD results.

**Figure 4:**
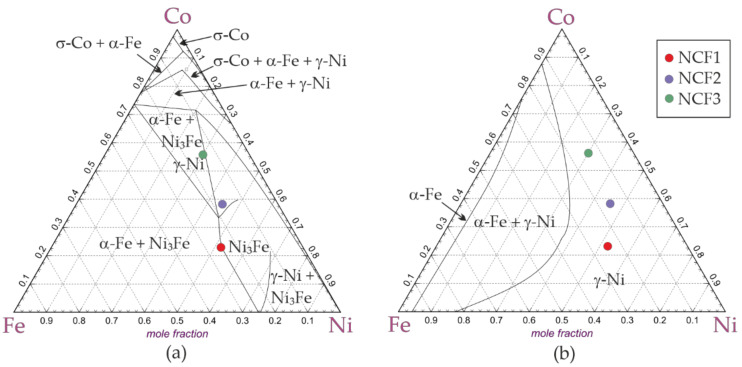
Isothermal cross-sections of the Ni-Co-Fe system for the temperature of (**a**) 50 °C and (**b**) 600 °C; chemical compositions of NCF1, NCF2 and NCF3 coatings are marked by dots.

The phase composition of the coatings was analyzed in terms of the equilibrium phases determined by the thermodynamic calculations. Figure 4 shows the isothermal cross-sections of the Ni-Co-Fe system for the temperature of 50 °C and 600 °C generated using the FactSage package. The thermodynamically stable phases predicted in equilibrium conditions at a temperature of 50 °C, at which the coatings were deposited, and at an elevated temperature of 600 °C are summarized in Table 4. The results of phase analysis are consistent with the results of thermodynamic calculations for a temperature of 600 °C, at which the γ-Ni is the stable phase for the chemical compositions of the NCF1, NCF2, and NCF3 coatings. Whereas at 50 °C, the other phases appeared. The above comparison leads to the conclusion that the electrocrystallization process of the coatings was similar to conventional crystallization of the liquid metal under a high cooling rate, which is a characteristic feature of this process [22].

Zhang and Ivey [32] also reported the single γ phase structure in Ni-Co-Fe alloys, characterized by similar chemical composition as those obtained in this study, while for higher Fe content of 29–69%, the formation of mixed γ and α phase structure was observed.

### 3.4. Microstructure

**Figure 5:**
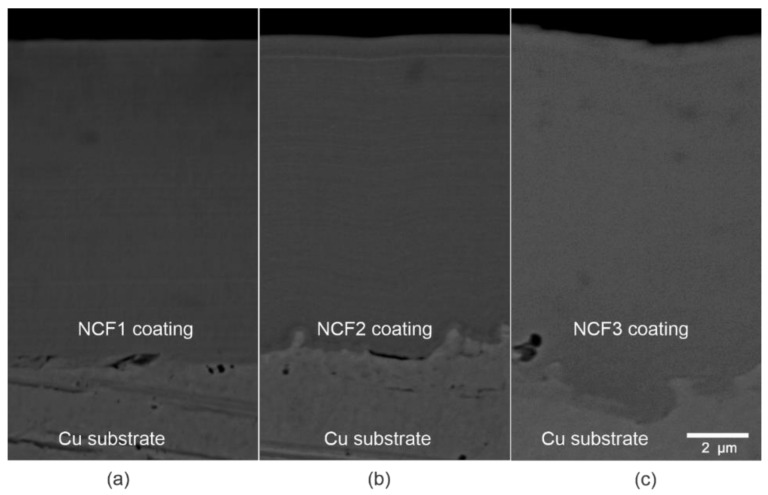
Cross-sections of the Ni-Co-Fe coatings: (**a**) NCF1, (**b**) NCF2, (**c**) NCF3, SEM BSE images.

Figure 5 shows typical cross-sectional low-magnification SEM BSE images of electrodeposited Ni-Co-Fe coatings. The thickness of the coatings was in the range of 8–11 µm. All coatings were homogenous, free of cracks and pores, and well adherent to the substrate. SEM observation of the smooth surface in the cross-section of the coating is in good agreement with the results of the roughness measurements.

**Figure 6:**
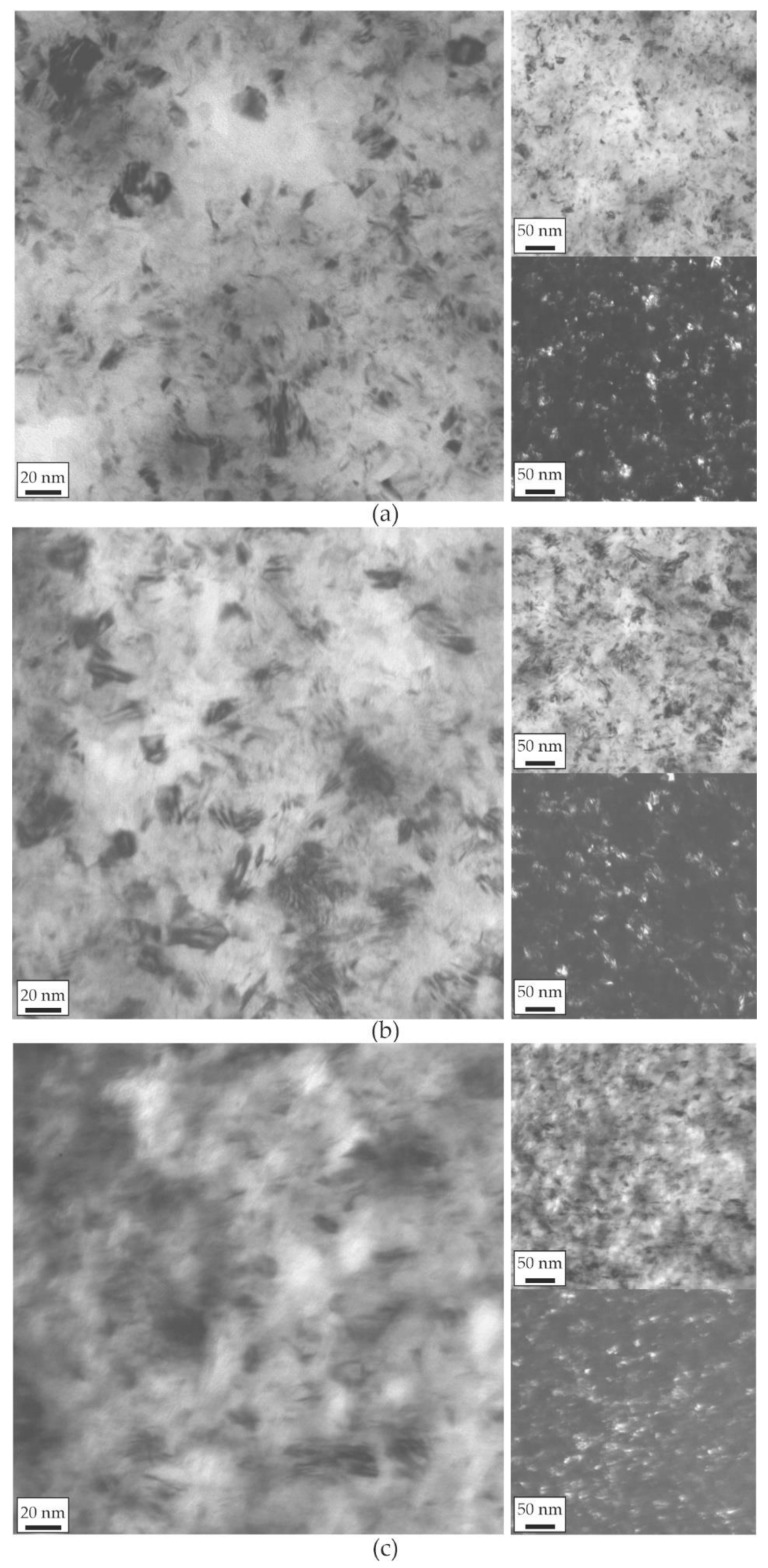
TEM bright-field images of (**a**) NCF1, (**b**) NCF2, and (**c**) NCF3 coatings and at the right the pairs of the lower magnification bright- and dark-field images taken in (111) ring of the γ phase.

Figure 6 shows TEM images of the Ni-Co-Fe coatings’ microstructure, consisting of equiaxed nanocrystalline grains with frequently occurring twins, which is typical for Ni-based electrolytic coatings [33,34].

**Figure 7:**
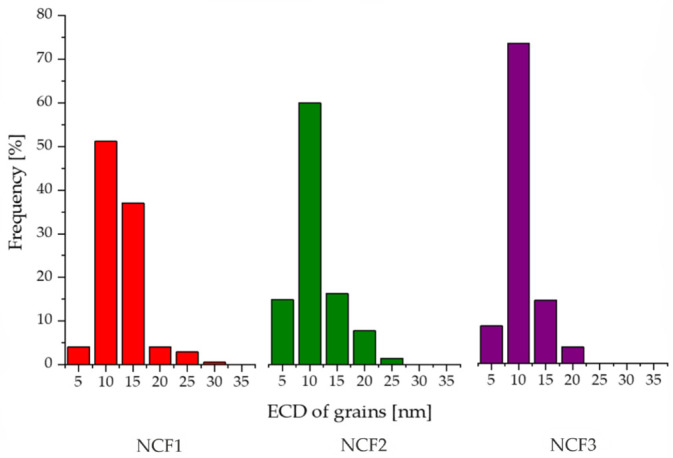
Grain size distribution in NCF1, NCF2, and NCF3 coatings.

Histograms of grain diameters are presented in Figure 7. In turn, results of grain size and mean crystallite size measurements performed by TEM image analysis and XRD are collected in Table 5. The values of the mean grain diameter $\bar{\mathrm{ECD}}$ and its standard deviation are equal to 10 ± 4 nm, 8 ± 4 nm, and 8 ± 3 nm for NCF1, NCF2, and NCF3, respectively. The diameters of the largest grains did not exceed 30 nm. The grain size for different coatings was in a similar range. The upper range of ECDs was lower for the NCF3 coating, which was characterized by the higher Co concentration.

Crystallite sizes calculated from the Scherer equation were in good agreement with the grain diameters determined with the use of TEM dark-field images. The calculated average crystallite size was in the range of 11–12 nm and was much lower than those obtained in Ni-Co-Fe coatings deposited from sulfate and chloride baths [11] or a modified Watts bath [5]. The grain size refinement might be related to the chemical composition of the electrolytic bath, particularly the addition of citrates and saccharin, which is in line with the results of Zhang and Ivey [32] obtained for electrodeposited Ni-Co-Fe coatings.

A decrease in grain size caused by increased alloying element concentration was also reported in other Ni-based electrodeposited alloys [35,36]. According to different Ni, Fe, and Co atom radius, an increase in alloying element content causes lattice distortion, which results in a higher probability of defect formation, such as dislocations and vacancies, which are nucleation sites of new grains.

Moreover, our ternary Ni-Co-Fe coatings were characterized by smaller grains than binary Ni-Co and Ni-Fe alloys electrodeposited from citrate-sulfate baths [27,28]. It is probably due to the presence of two alloying elements in a nickel matrix and the addition of saccharin. The grain refinement effect of saccharin in electrodeposited Ni coating was described by El-Sherik [37] and Rashidi et al. [38]. They explained it by increasing the nucleation rate of new grains and the accompanying formation of complex compounds that inhibit diffusion of metal ions, resulting in blocking grain growth.

In all Ni-Co-Fe coatings, the microstructure consisted of equiaxed grains. Only a low number of elongated grains was noticed, which indicates that columnar grain growth, a characteristic of electrodeposited Ni-based coatings, was disturbed by organic additives [28,33,39]. Similar results were reported by Kolonist et al. in Ni coatings electrodeposited from Watts bath in the presence of saccharin [39].

### 3.5. Microhardness

The results of microhardness measurements are collected in Table 6. Electrodeposited coatings were characterized by a high hardness above 4.2 ± 0.4 GPa. The obtained values are in the range typical for electrodeposited Ni-based alloys [28,40,41]. The highest hardness of NCF2 coating may be related to both the smallest grains and the solid solution strengthening. In MEMS application, hard coatings are characterized by better wear and scratch resistance; therefore, a high microhardness is recommended [42].

It is well known that the hardness of materials enhances with the refinement of grains because of an increase in the volume fraction of grain boundaries, which impede the dislocations’ movement [43]. However, in nanocrystalline materials, a decrease in hardness was observed for grain refinement below a critical value. For example, Wu et al. [44] reported a decrease in hardness for Ni-Co alloys with crystallites smaller than 15 nm. In this work, the mean size of grains/crystallites in electrodeposited ternary alloys was in the range of 8–12 nm. It suggests that the hardness increase with the grain refinement should no longer be observed. Therefore, the increase in hardness may be associated with the solution hardening. It was reported that in binary Ni-Co alloys, hardness increases for Co content up to 45–50 wt.%, and drops for higher concentration [45,46,47]. In binary Ni-Fe alloys, the mechanism of hardening is challenging to determine because grain refinement is observed with an increase in Fe content. Sanaty-Zadeh et al. [48] and Guo et al. [49] suggested that the hardening is achieved mainly by grain refinement, while the solid solution hardening has only a minor effect.

In ternary Ni-Co-Fe alloys, the solution hardening is more complex than in binary Ni-Co and Ni-Fe systems. According to the results of Divya et al. [50], the peak of hardness occurs at a particular composition approximate to Ni_38_Co_35_Fe_27_. This composition is similar to that of the NCF2 coating (Ni_49_Co_37_Fe_17_). On this basis, it can be concluded that the hardness of this coating, higher than that of the others, results from grain refinement and solid solution strengthening.

### 3.6. Magnetic Properties

**Figure 8:**
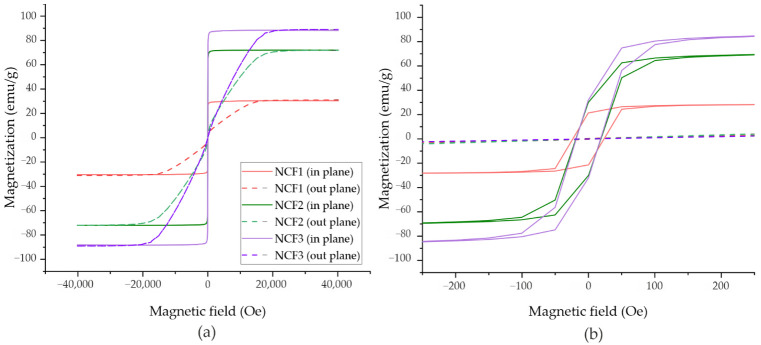
(**a**) Magnetic hysteresis loops measured at room temperature for ternary Ni-Co-Fe alloys shown in the full range and (**b**) at the small field area, in-plane (solid line), and out-of-plane (dash line) geometry.

Magnetic properties of ternary Ni-Co-Fe coatings were measured using a SQUID magnetometer (MPMS, Quantum Design North America, San Diego, CA, USA). Ni, Fe, and Co are ferromagnetic elements, and their magnetic saturation is equal to 55.1 emu/g [51], 217.6 emu/g [51], and 168.0 emu/g [52], respectively. The magnetic hysteresis loops with the magnetic field applied in-plane and out-of-plane are presented in Figure 8, while magnetic parameters are collected in Table 7. The measurements indicate high magnetic anisotropy of all Ni-Co-Fe coatings with an easy axis of magnetization in the surface plane. The coercivity values determined from in-plane measured hysteresis loops showed small H_c_ in the range of 19–23 Oe. In in-plane geometry, the coatings reached the saturation of magnetization at fields about 0.2 kOe, while in out-of-plane measurements at 20 kOe. The saturation magnetization M_s_ changed from 31.0 to 88.1 emu/g for coatings with the highest and lowest Co concentration. NCF2 and NCF3 samples were characterized by both higher M_s_ and lower H_c_ than NCF1.

Magnetic properties of electrodeposited coatings are influenced by many factors, such as bath composition, temperature, or electrodeposition current parameters, which also affect the microstructure, chemical and phase composition, or internal stresses. However, M_s_ depends mainly on the chemical composition of the coatings. Our results showed that the increase in Co content in ternary alloys caused a significant rise in M_s_ due to the replacement of some of Ni atoms by Co atoms, characterized by a higher magnetic saturation.

The coercivity is strongly related to the microstructure and size of magnetic domains, which in polycrystalline materials might be assumed as identical with the size of crystallites/grains. H_c_ increases for magnetic domains bigger or smaller than magnetic exchange length parameter (L_ex_), which for Fe, Ni, and Co is 14 nm, 70 nm, and 55 nm, respectively [53]. NCF2 and NCF3 coatings were characterized by a lower H_c_ than NCF1, which can be related to the lower number of grains with diameters larger than 20 nm (Figure 7).

The deposited alloys exhibit different magnetic properties than those described by Osaka et al. [16], mainly due to the different chemical compositions. According to the diagram of magnetic properties presented in [1], lower Fe and higher Ni contents than for Osaka’s alloy [16] provided to obtain higher coercivity and lower saturation magnetization.

The magnetic coercivity of the NCF1, NCF2 and NCF3 coatings is comparable to that obtained in similar coatings studied by Zhang and Ivey [32] and Yoo et al. [24] and lower than reported by Kuru et al. [5] and Ismail et al. [54].

The achieved results show that the deposited coatings exhibit soft magnetic properties suitable for prospective magnetic MEMS applications.

### 3.7. Corrosion Properties

**Figure 9:**
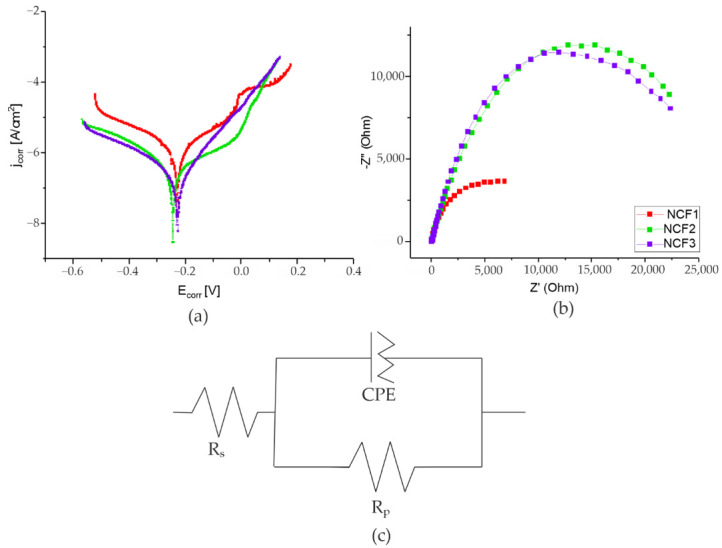
(**a**) Polarization curves, (**b**) Nyquist plots of ternary Ni-Co-Fe curves, (**c**) Randles equivalent circuit (R_s_—solution resistance, R_p_—charge transfer resistance, CPE—double layer capacity) obtained for Ni-Co-Fe coatings.

Measurements of the corrosion properties were performed in a 2.0% NaCl solution. Polarization curves and results of EIS measurements are shown in Figure 9, while calculated corrosion parameters are collected in Table 8. Results of the EIS measurements were in good agreement with those obtained in the polarization tests. The coatings were characterized by a good corrosion resistance in chlorine media. It was observed that the corrosion current of NCF2 and NCF3 coating was lower than for NCF1. Moreover, NCF1 was characterized by more electronegative potential E_corr_ than NCF2 and NCF3, probably due to higher Fe concentration. For NCF1 and NCF2, the passive area was present, which might be related to high Ni content [4].

The fitting of experimental data with the Randles circuit showed a higher polarization resistance of NCF2 and NCF3 than for NCF1 coating, which is also in line with polarization tests.

The influence of chemical composition and microstructure on the corrosion resistance of the coatings is crucial. Likely, the more compact structure and smaller grain size observed in NCF2 and NCF3 coatings have an influence on the higher R_p_ and corrosion resistance of the coatings.

The effect of Fe and Co content on corrosion resistance was analyzed based on literature reports on binary Ni-Fe and Ni-Co coatings. In Ni-Fe alloys, the corrosion resistance decreases with increasing Fe concentration [55]. In turn, the corrosion resistance of Ni-Co alloys is better than that of pure Ni up to the content of 38% Co, with the maximum for 17%, and above 38% Co is worse [56].

The literature on the corrosion resistance of Ni-Co-Fe coatings is limited. Yoo et al. [24] reported the results obtained for electrodeposited Ni_48_Co_36_Fe_16_ alloy, with a composition similar to NCF2. They showed that such coating was characterized by higher R_p_ and lower pitting potential than those containing lower or higher Ni content. They found that higher corrosion resistance was a consequence of a fine-grain and single-phase fcc structure, which is consistent with our results.

## 4. Conclusions

In our study, we electrodeposited ternary Ni-Co-Fe coatings with three different chemical compositions and investigated the influence of their microstructure, chemical and phase composition on microhardness, magnetic properties, and corrosion resistance. The deposited materials offer a good combination of properties, and after further development, they could be potentially used as magnetic sensors or microactuators. Based on the results, the following conclusions are reached:

- Electrodeposition of Ni-Co-Fe coatings from the citrate-sulfate bath is anomalous, and ions are reduced in the Fe^2+^, Co^2+^, and Ni^2+^ order. Reduction rates of Fe^2+^ and Co^2+^ ions are significantly higher than of Ni^2+^.
- The surface morphology of Ni-Co-Fe coatings is smooth with very small nodules. Only a low number of surface defects, such as irregularly shaped islands, porosity or discontinuities, were noticed.
- The microstructure of electrodeposited coatings consists of nanometric, equiaxed grains. The mean grain diameters measured in TEM dark-field images and mean crystallite sizes determined by XRD are in the range from 8 nm to 12 nm.
- Both XRD and SAED diffraction analysis confirmed that the microstructure of ternary Ni-Co-Fe coatings consists of a single fcc γ phase. It proves that the phase composition of the deposited coatings deviates from the equilibrium and is in good agreement with thermodynamic simulations for a temperature of 600 °C, at which the γ-Ni is the stable phase.
- Ni-Co-Fe coatings are characterized by high hardness above 4.2 ± 0.4 GPa.
- The Ni-Fe-Co coatings exhibit required soft magnetic properties with coercivity below 23 Oe.
- The corrosion resistance of Ni-Co-Fe coatings is satisfactory. It was confirmed that the higher Fe content leads to the deterioration of the corrosion resistance.

The nanocrystalline structure, high hardness, good corrosion resistance, low coercivity, and relatively large magnetization saturation make Ni-Fe-Co coatings promising materials for MEMS applications.

## Figures and Tables

**Table 1:** Parameters of electrodeposition and chemical composition of electrolytic baths.

		Bath	
Compound (g/L)	NCF1	NCF2	NCF3
NiSO_4_ · 6H_2_O	210	210	210
FeSO_4_ · 6H_2_O	15	15	15
CoSO_4_ · 6H_2_O	15	30	60
sodium citrate (Na_3_C_6_H_5_O_7_)	30	30	30
ascorbic acid	1	1	1
SLS	0.5	0.5	0.5
sodium saccharin	1	1	1
**Parameters**			
current density (A/dm^2^)	2.5		
deposition time	1200		
temperature (°C)	50		
distance between electrodes (mm)	10		
pH	4.0 ± 0.1		

**Table 2:** Roughness parameters of ternary Ni-Co-Fe alloys: arithmetical mean deviation of the assessed profile R_a_, root mean squared roughness R_q_, and a maximum height of the profile R_t_.

Parameter	NCF1	NCF2	NCF3
R_a_ (µm)	0.21	0.09	0.14
R_q_ (µm)	0.28	0.13	0.20
R_t_ (µm)	6.17	5.87	6.38

**Table 3:** The results of the XRD measurements.

No.	h	k	l	Pattern [12]		NCF1		NCF2		NCF3	
				2theta	Relative Intensity	2theta	Relative Intensity	2theta	Relative Intensity	2theta	Relative Intensity
1	1	1	1	44.086	100.0	44.05	100.0	44.175	100.0	44.44	100.0
2	2	0	0	51.362	42.9	51.17	24.8	51.301	25.8	51.69	105.6
3	2	2	0	75.593	17.2	75.58	9.5	-	-	-	-
4	3	1	1	91.883	15.8	91.94	11.8	91.811	9.1	92.47	9.1
5	2	2	2	97.291	4.3	97.35	8.5	-	-	-	-

**Table 4:** The thermodynamically stable phases predicted for the chemical compositions of the NCF1, NCF2, and NCF3 coatings in equilibrium conditions at 50 °C and 600 °C.

Coating	NCF1	NCF2	NCF3
Temperature	Thermodynamically Stable Phases		
50 °C	α-Fe ^1^, Ni_3_Fe ^2^	Ni_3_Fe + γ-Ni ^3^	α-Fe, Ni_3_Fe + γ-Ni
600 °C	γ-Ni	γ-Ni	γ-Ni

^1^ Fe-based solid solution with body-centered cubic (bcc) structure, ^2^ L12 intermetallic phase, an ordered counterpart of fcc solid solution, ^3^ Ni-based solid solution with fcc structure.

**Table 5:** The results of grain size and mean crystallite size measurements performed by TEM image analysis and XRD.

Coating	Grain Size Determined by TEM		Average Crystallite Size Estimated by XRD
	ECD Range (nm)	$\bar{\mathrm{ECD}}$ ± s (nm)	d (nm)
NCF1	4–27	10 ± 4	12 ± 1
NCF2	3–25	8 ± 4	11 ± 1
NCF3	2–20	8 ± 3	12 ± 1

**Table 6:** The microhardness of ternary Ni-Co-Fe coatings.

	NCF1	NCF2	NCF3
Hardness (GPa)	4.4 ± 0.4	4.6 ± 0.3	4.2 ± 0.4

**Table 7:** Magnetic properties of ternary Ni-Co-Fe coatings: saturation M_s_, remanence M_r_, and coercivity H_c_.

Coating	Chemical Composition	M_s_ (emu/g)	M_r_ (emu/g)	H_c_ (In-Plane) (Oe)	H_c_ (Out-of-Plane) (Oe)
NCF1	Ni_52_Co_23_Fe_25_	31.0	21	23	0
NCF2	Ni_46_Co_37_Fe_17_	72.0	31	19	0
NCF3	Ni_30_Co_56_Fe_14_	88.1	30	19	0

**Table 8:** Corrosion properties of ternary Ni-Co-Fe coatings: corrosion current density j_corr_, corrosion rate V_corr_, corrosion potential E_corr_, impedance value for 0.1 Hz|Z|, solution resistance R_s_, charge transfer resistance R_p_, double layer capacity Q_0_>, and constant phase n.

Coating	j_corr_ (A/cm^2^)	V_corr_ (mm/year)	E_corr_ vs. Ag/AgCl (V)	|Z| 0.1 Hz (Ω/cm^2^)	R_s_ (Ohm)	R_p_ (kOhm)	Q_0_ (F)	n
NCF1	2.5 × 10^−6^	1.11 × 10^−3^	−0.29	3866	14	9.08	7.5 × 10^−5^	0.99
NCF2	2.6 × 10^−7^	1.16 × 10^−4^	−0.24	11965	13	30.6	1.0 × 10^−5^	0.99
NCF3	3.7 × 10^−7^	1.66 × 10^−4^	−0.23	11896	25	27.5	1.0 × 10^−5^	0.99

## Data Availability

The data presented in this study are available at the Corresponding authors.
